# Quality of anticoagulation and use of warfarin-interacting medications in long-term care: A chart review

**DOI:** 10.1186/1471-2318-8-13

**Published:** 2008-07-03

**Authors:** Madeleine Verhovsek, Bahareh Motlagh, Mark A Crowther, Courtney Kennedy, Lisa Dolovich, Glenda Campbell, Luqi Wang, Alexandra Papaioannou

**Affiliations:** 1 McMaster University, Hamilton, Ontario, Canada; 2 Medical Pharmacies Group Inc., Pickering, Ontario, Canada; 3 St. Joseph's Healthcare, Hamilton, Ontario, Canada

## Abstract

**Background:**

Maintenance of therapeutic International Normalized Ratio (INR) in the community is generally poor. The supervised environment in long-term care facilities may represent a more ideal setting for warfarin therapy since laboratory monitoring, compliance, dose adjustment, and interacting medications can all be monitored and controlled. The objectives of this study were to determine how effectively warfarin was administered to a cohort of residents in long-term care facilities, to identify the proportion of residents prescribed warfarin-interacting drugs and to ascertain factors associated with poor INR control.

**Methods:**

A chart review of 105 residents receiving warfarin therapy in five long-term care facilities in Hamilton, Ontario was performed. Data were collected on INR levels, warfarin prescribing and monitoring practices, and use of interacting medications.

**Results:**

Over a 12 month period (28,555 resident-days, 78.2 resident years) 3065 INR values were available. Residents were within, below and above the therapeutic range 54%, 35% and 11% of the time, respectively. Seventy-nine percent of residents were prescribed at least one warfarin-interacting medication during the period in review. Residents receiving interacting medications spent less time in the therapeutic range (53.0% vs. 58.2%, OR = 0.93, 95% confidence interval 0.88 to 0.97, P = 0.002). Adequacy of anticoagulation varied significantly between physicians (time in therapeutic range 45.9 to 63.9%).

**Conclusion:**

In this group of long-term care residents, warfarin control was suboptimal. Both prescriber and co-prescription of interacting medications were associated with poorer INR control. Future studies should seek strategies to improve prescriber skill and decrease use of interacting medications.

## Background

Warfarin is frequently prescribed to the elderly; often for primary or secondary prevention of stroke in patients with atrial fibrillation [1] or to prevent recurrent venous thromboembolism (VTE) [2] Residents of long-term care (LTC) facilities are particularly likely to receive warfarin given the high prevalence of atrial fibrillation [3,4] and incidence of VTE [5] in this older population.

Although good quality evidence suggests an optimal target International Normalized Ratio (INR) of 2.0–3.0 for these common indications [6-8], maintenance of therapeutic INR in the community is generally poor [9-11]. LTC facilities may represent ideal environments for warfarin therapy since laboratory monitoring, compliance, dose adjustment, and interacting medications can all be monitored and controlled.

Despite the frequency of warfarin use in LTC and the unique care environment, relatively few studies have examined how well warfarin therapy is provided to residents of LTC facilities. We sought to address this knowledge deficit by performing a retrospective chart review. Our objectives were to determine: (1) the percentage of time residents spent in the therapeutic international normalized ratio (INR) range, (2) the prevalence and incidence of prescription of warfarin-interacting drugs and (3) whether co-prescription of medications known to interact with warfarin was associated with the proportion of time in the therapeutic range.

## Methods

### Setting and patients

The study sample consisted of all residents at five LTC facilities in Hamilton, Ontario. The facilities had a total of 1144 residents (mean 229 residents). These facilities provide nursing care and residential facilities for individuals no longer able to live in the community; each facility provides medication dispensing and laboratory monitoring services in addition to assistance with activities of daily living. All prescription medications received by residents are dispensed by a single pharmacy providing service to that facility.

### Study design

Residents were identified from the centralized pharmacy database for the LTC facilities. The retrospective chart review included data from the preceding twelve months. Using a standardized form, two reviewers (MV and BM) abstracted data from facility charts for each resident receiving warfarin for the period between October 2004 and April 2005. Information collected included: indication for anticoagulation, INR results, target range, and INR-testing intervals. In addition demographic data for each participant, including age, sex, comorbidities, concomitant medications and prescribing physician was recorded for statistical analysis.

To identify prescription of medications known to interact with warfarin, we compiled a list based on "highly probable" and "probable" interactions in a recent systematic review, [12] and cross-referenced it with the pharmacy database for our sample population. Initiation or change in dose of medications known to interact with warfarin was also recorded.

### Statistical analysis

Analysis of quantitative (including descriptive statistics) and qualitative data was conducted. In the primary analysis we assessed quality of treatment based on TTR (therapeutic range = 2.0–3.0). Linear interpolation of INR, in the method of Rosendaal et.al. [13], was used to characterize each day of warfarin therapy: INR values were assigned to each day between INR measurements based on a postulated linear change. Values were rounded to one decimal place. Mantel-Haenszel weighted odds ratios (OR's) and 95% CI's were computed where appropriate (EpiInfo, Centers for Disease Control, Atlanta, Georgia, 2004). The association between baseline and time dependent factors and adequacy of anticoagulation was to be queried using univariable analysis. Multivariable analysis proved to not be possible because of strong interaction between baseline and time dependent variables.

### Ethics

Before proceeding with data collection, approval was obtained from the Medical Director, Administrator, and Director of Care at each facility. Consent was not obtained from individual residents. Each resident was assigned a numerical code which was used on all study documents to prevent identification of their personal information. The facility's Professional Advisory Committee reviewed the protocol. Approval for this project was received from the Research Ethics Board at McMaster University.

## Results

### Sample demographics

Overall, 107 of 1144 (9%, 95% CI 8% to 11%) residents were prescribed warfarin. Residents prescribed warfarin were predominantly female (72%), with a mean age of 83.6 years (range 54.7–98.0). All but two of the residents were over the age of 65. Two residents had medical indications for target INR above 2.0–3.0 and were thus excluded from the analysis. Depending on length of stay at the LTC facilities and duration of anticoagulation with warfarin, the duration of the audit for individual residents ranged from 0.7 to 13.3 months (mean 9.1).

**Figure 1:**
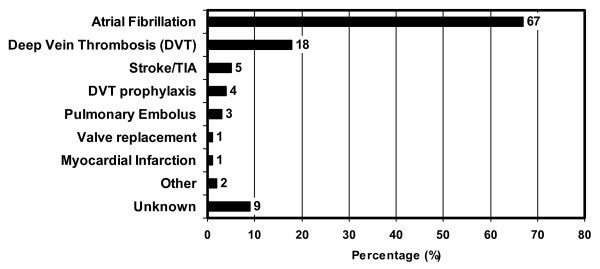
**Indications for warfarin**. Indications for warfarin included as other: Sick sinus syndrome and peripheral vascular disease.

The majority of residents in our sample had atrial fibrillation (67%), or a history of stroke or transient ischemic attack (53%). A large proportion had hypertension (50%), coronary artery disease (48%), dementia (37%), osteoarthritis (31%), congestive heart failure (31%) or diabetes mellitus (22%). The percentage of residents with depression, chronic obstructive pulmonary disease, osteoporosis, asthma, anemia, renal impairment and history of cancer was 18%, 14%, 14%, 10%, 8%, 6% and 20% respectively. The most common indications for warfarin were stroke prophylaxis in atrial fibrillation (67%) and treatment of deep vein thrombosis (18%) (Figure 1). In 9 of the 105 residents (9%) the indication for anticoagulation with warfarin was not clear after complete review of the facility chart, though the stated target INR was 2.0–3.0. Ten residents had two documented indications for warfarin therapy.

### INR

**Figure 2:**
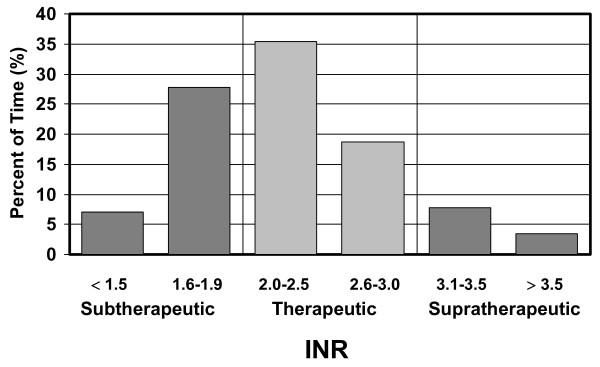
**Time-in-therapeutic range**. Over 28,555 resident-days, time spent in specific INR ranges.

Over the period of the chart review, a total of 3065 INR values were available, representing 28,555 resident days (78.2 resident years). The average number of INR measurements per resident was 3.4 per month (average of one measurement every 9 days). Linear interpolation of INR values [13] was performed to assign an INR value to each patient day. Overall, residents spent 54.1% of time in the therapeutic range (INR 2.0–3.0). Residents' anticoagulation was subtherapeutic (<2.0) 34.7% of the time and supratherapeutic (>3.0) 11.2% of the time. We further broke down the data into ranges and found that a large proportion of the time (27.7%) was spent in the INR range of 1.6–1.9 (Figure 2).

### Interacting medications

Overall, 79% of residents on warfarin therapy (83 residents) were prescribed at least one interacting drug during the period of the chart review (mean no. interacting medications = 1.8, range 1–6). The five most common drugs were acetaminophen, citalopram, acetylsalicylic acid, diltiazem and simvastatin (Table 1). To examine monitoring practices, we further determined whether addition or dose change of drugs known to interact with warfarin was followed by INR measurement within seven days. There were 72 instances of newly-initiated warfarin-interacting medications or changes in dose. In 59 of 72 instances (82%) the INR was checked within 7 days of the initiation of the medication.

**Table 1:** Prevalence of interacting medications. Number (percentage) of residents prescribed each warfarin-interacting medication over the period of the chart audit.

**Medication**	**No. of Residents Prescribed (%)**
Acetaminophen	42 (40%)
Citalopram	26 (25%)
Acetylsalicylic acid	17 (16%)
Diltiazem	12 (11%)
Simvastatin	10 (10%)
Levofloxacin	8 (8%)
Phenytoin	7 (7%)
Ciprofloxacin	5 (5%)
Sertraline	5 (5%)
Cotrimoxazole	3 (3%)
Metronidazole	3 (3%)
Clarithromycin	3 (3%)
Amiodarone	3 (3%)
Amoxicillin-clavulanate	2 (2%)
Miconazole	1 (1%)
Propranolol	1 (1%)
Fluvoxamine	1 (1%)

Medications not prescribed: erythromycin, fluconazole, fluvastatin, quinidine, ropinerole, celecoxib and entacapone.

Residents receiving warfarin-interacting medications during the period of the chart audit had a TTR of 53.0%, compared with a TTR of 58.2% in the residents on no interacting drugs (OR = 0.93, 95% CI, 0.88 to 0.97, P = 0.002). Residents with 2 or more interacting medications had a lower TTR (50.8%) than those with only one interacting medication (55.1%) (Table 2a).

**Table 2:** TTR calculated for variables of a) interacting medications and b) prescribing physician

a)				
Warfarin-Interacting Medications?	Number of Interacting Meds	Number of INR-Days	Days in Therapeutic Range (INR 2.0–3.0)	TTR (%)

**Yes**	Overall	22683	12028	**53.0**
	3+	4328	2199	50.8
	2	6608	3357	50.8
	1	11747	6472	55.1
**No**	None	5872	3419	**58.2**

b)				

Physician ID#		Number of INR-Days	Days in Therapeutic Range (INR 2.0–3.0)	TTR (%)

1		2150	1360	63.3
2		3137	1967	62.7
3		1468	916	62.4
4		2310	1396	60.4
5		1083	619	57.2
6		3334	1823	54.7
7		971	474	48.8
8		6144	2961	48.2
9		700	337	48.1
10		4383	2011	45.9

### Prescribing physicians

Twenty individual physicians were identified as warfarin prescribers at the five facilities (prescribers-per-facility range, 2–10). To increase the reliability of our observations, TTR was determined for only the ten physicians with (1) three or more patients receiving warfarin and (2) total patient-INR-days ≥ 700. We found significant differences in the achieved TTR between the physicians (range 45.9% to 63.3%, OR = 1.06, 95% CI, 1.00 to 1.13, p = 0.05, Table 2b).

## Discussion

This study is the first to assess TTR in LTC residents on warfarin in Canada. We found that the quality of anticoagulant care was sub-optimal; overall, INR was in the therapeutic range 54% of the time and was subtherapeutic 35% of the time. The majority of residents received at least one warfarin-interacting drug. Eighty-two percent of times an interacting drug was started, INR testing occurred within one week, which compares favorably to monitoring in a study in outpatients (INR testing within 14 days, 77% of the time) [14]. Despite monitoring, TTR was lower in residents receiving warfarin-interacting medications. The quality of anticoagulation also varied significantly between physicians (TTR 45.9 to 63.3%).

The overall TTR result we obtained is comparable with that reported in three previous studies (TTR range 40 to 51%) [15-17]. However, the values compare unfavorably with an average TTR of 61% in studies in outpatients receiving warfarin. [6]

The finding that patients in LTC facilities have suboptimal INR control is surprising given the fact that many conditions which predict poor control in the community (in particular lack of compliance, failure to account for interacting medications, and failure to undergo monitoring) should be controlled in LTC. In fact each of these variables was controlled in our study; patient compliance was assured by central medication dispensing, monitored administration and frequent monitoring [18,19]. In addition to frequent use of interacting drugs in a LTC setting, other barriers to optimal INR control may include frequent staff shift changes, communication failures regarding dose adjustment and resident mental health issues.

Our study has several limitations including a limited sample size. The chart audit design did not allow us to track the possible combined effect of interacting medications and warfarin doses on INR results, limited our ability to explore additional factors that may have contributed to sub-optimal INR such as acute co-morbidities and variability in dietary vitamin K intake and also prevented multivariable analysis. In addition, we did not verify administration of individual warfarin doses. Despite this our results are important as we used rigorous methods to review, in duplicate, clinical charts from a well characterized cohort of patients from a LTC facility. We carefully calculated the TTR, and used rigorous statistical methodology to explore factors that may have contributed to inadequate anticoagulant control.

## Conclusion

Our study provides preliminary evidence of the high prevalence of co-prescription of warfarin and interacting medications in LTC and the correlation with poorer INR control. The area of co-prescription of warfarin and interacting drugs has been inadequately studied – in the community and in LTC – and, to our knowledge, this study represents the first study of its kind. As suggested by a recent study in ambulatory care [20], a combined approach of physician education and automated alerts may be useful in decreasing co-prescription of interacting drugs. If a warfarin-interacting drug was medically necessary for a patient receiving oral anticoagulation, this approach, combined with pharmacy monitoring, would encourage prompt post-prescription INR monitoring.

Our study was implemented after completion of a learning needs-assessment with the medical directors of the five facilities. The results presented here have been shared with all the directors of care who support a planned future study assessing methods to improve TTR and drug-INR monitoring in long-term care. This trial will compare a previously validated decision-support tool [21] and automated interactions alerts with traditional physician management after academic detailing by a pharmacist on appropriate target INR and interaction management. Future studies should examine several variables simultaneously to determine which are most predictive of improved TTR.

## Competing interests

The authors declare that they have no competing interests.

## Authors' contributions

MV and BM contributed to concept and design of study; data gathering, analysis and interpretation; and preparation of article for submission. MAC, CK, LD, GC, LW and AP contributed to concept and design of study; data analysis and interpretation; and preparation of the manuscript for submission. All authors read and approved the final manuscript.

## Pre-publication history

The pre-publication history for this paper can be accessed here:


